# Interactions between Bitter Taste Receptor Gene Variants and Dietary Intake Are Associated with the Incidence of Type 2 Diabetes Mellitus in Middle-Aged and Older Korean Adults

**DOI:** 10.3390/ijms24032199

**Published:** 2023-01-22

**Authors:** Kyung Won Lee, Dayeon Shin

**Affiliations:** 1 Department of Home Economics Education, Korea National University of Education, Cheongju 28173, Republic of Korea; 2 Department of Food and Nutrition, Inha University, Incheon 22212, Republic of Korea

**Keywords:** *TAS2R4* gene variants, gene–diet interaction, cohort study, type 2 diabetes mellitus, Korean adults

## Abstract

The relationship between the variants of bitter taste receptor gene *TAS2R4*, dietary intake, and incidence of type 2 diabetes mellitus (T2DM) remains unclear. Hence, we aimed to examine the association of *TAS2R4* rs2233998 variants with T2DM incidence in middle-aged and older Korean adults to understand if their association was modulated by dietary intake. Data of the Ansan-Ansung cohort from the Korean Genome and Epidemiology Study were used in this study. A total of 4552 Korean adults aged 40–69 years with no history of T2DM or cancer at baseline were followed-up for 16 years. Dietary intake was assessed using a 103-item food frequency questionnaire, and new T2DM cases were defined based on the World Health Organization and International Diabetes Federation criteria. Multivariate Cox proportional hazards models were used to estimate hazard ratios (HRs) and 95% confidence intervals (CIs) for T2DM incidence. During the mean follow-up period of 11.97 years, 1082 (23.77%) new T2DM cases were identified. Women carrying the TT genotype of *TAS2R4* rs2233998 exhibited 1.48 times higher incidence of T2DM (HR: 1.48; 95 CI: 1.13–1.93) than those carrying the CC genotype. *TAS2R4* rs2233998 variants were positively associated with the incidence of T2DM among Korean women with high intakes of carbohydrates or sugars and low intakes of fruits or vegetables. TT carrier women in the highest tertile of carbohydrate or sugar intake exhibited an increased incidence of T2DM (HR: 2.08, 95% CI: 1.33–3.27 for carbohydrates; HR: 2.31, 95% CI: 1.53–3.51 for sugars) than CC carrier women. Women carrying the TT genotype in the lowest tertile exhibited an increased incidence of T2DM (HR: 1.55, 95% CI: 1.02–2.37 for vegetables; HR: 1.62, 95% CI: 1.06–2.48 for fruits) than women carrying the CC genotype in the highest tertile of vegetable or fruit consumption. However, no association was observed between *TAS2R4* rs2233998 variants and dietary intake with T2DM incidence in Korean men. Our findings suggest that variants of *TAS2R4* rs2233998 are associated with T2DM incidence, and their associations are strengthened by excessive intake of carbohydrates or sugars and inadequate intake of fruits or vegetables. Diet encompassing optimal intake of carbohydrates or sugars and high intake of fruits or vegetables may minimize the risk of developing T2DM.

## 1. Introduction

Diabetes is a chronic condition characterized by consistently high blood glucose levels because of insufficient production of insulin by the body or the ineffectiveness of the produced insulin. Globally, 537 million adults (9% prevalence) have been diagnosed with diabetes, and it is expected that this number will increase to 783 million by 2045, i.e., a 46% increase [1]. A study on disease burden in Korea ranked diabetes as the eighth leading cause of death in 2019 [2], and it reported that one in seven (13.8%) Korean adults aged ≥30 years had diabetes as of 2018 [3]. The prevalence of diabetes was 10% or higher in both men and women aged 50 years or older, and its prevalence increases with age.

Diabetes can lead to other more serious complications such as cardiovascular diseases, neuropathy, and nephropathy; hence, prevention and management of diabetes is crucial. Diabetes is a multifactorial disease caused by interactions between environmental factors and genetic variants. Many studies have been conducted to identify risk factors associated with the development of diabetes. Previous genome-wide association studies (GWAS) identified the following genes related to diabetes: *TCF7L2*, *SLC30A8* (zinc transporter), *IDE-KIF11-HHEX*, and *EXT2-ALX4* in the French population [4] and *GLIS3*, *PEPD*, *FITM2-R3HDML-HNF4A*, *KCNK16*, *MAEA*, *GCC1-PAX4, PSMD6*, and *ZFAND3* in East Asian populations [5]. In the Korean population, CCDC63, C12orf51, and 2p21 gene variants were significantly associated with diabetes [6,7].

Recently, the taste receptor genes associated with diabetes have attracted the attention of the scientific community and are being investigated using GWAS to discover the susceptibility loci for type 2 diabetes mellitus (T2DM). Variants of taste receptor genes modify taste perception [8] that plays an important role in determining the food preferences, food choices, and eating behaviors of individuals [9]. Consequently, taste perception affects the nutritional and health status of an individual and can ultimately contribute to the risk of developing chronic diseases. Conversely, taste perception can change after the onset of a chronic disease. Fernández-Carrión et al. [10] observed alterations in taste perception and preference in individuals with an onset of diabetes. Individuals with diabetes exhibited lower perception of all tastes including bitter taste than those without T2DM. Moreover, individuals with T2DM exhibited a higher preference for sweet taste than individuals without T2DM. Among the five basic tastes, three (sweet, bitter, and umami) are detected by G protein-coupled receptors. Specifically, sweet, bitter, and umami tastes are distinguished by TAS1R2/TAS1R3, TAS1R1/TAS1R3, and TAS2R receptors, respectively. The sweet taste receptor gene variants have been associated with carbohydrate intake and hypertriglyceridemia in the Western population [11], and the relationships of bitter taste receptor genes with longevity [12] and diabetes [13] were also investigated. However, a few studies have reported inverse association between variants of the bitter taste receptor gene *TAS2R38* and the risk of gastric and colorectal cancers in Korean populations [14,15]. Although the relationship between the alterations in taste perception in patients with diabetes and that between taste receptor genes and T2DM parameters have been investigated, the effect of dietary intake on the relationship between taste receptor gene variants and T2DM development is unclear. Furthermore, it is important that these relationships are investigated in the Korean population because taste perception and preferences vary between cultures and ethnicities [16,17]. 

Therefore, in this study, we investigated the associations between the genotype of bitter taste receptor genes and T2DM to elucidate the role of taste receptor genes and their interactions with dietary intake in T2DM using a large cohort of the Korean population.

## 2. Results

### 2.1. General Characteristics of the Study Population

The general characteristics of the study participants (men and women) at baseline according to the *TAS2R4* rs2233998 genotype are shown in Table 1. Among the study population, 47.18%, 42.96%, and 9.86% of men had the CC, CT, and TT genotypes, respectively, and 43.74%, 45.55%, and 10.71% of women had the CC, CT, and TT genotypes, respectively. The women participants with the TT genotype were older than those carrying the C allele (CC: 51.9 ± 8.6 years, CT: 51.0 ± 8.4 years vs. TT: 52.3 ± 8.7 years; *p*-value < 0.05). No significant differences were found in the distribution of residential area, education level, smoking status, family history of diabetes, alcohol consumption, or mean body mass index (BMI) according to the *TAS2R4* rs2233998 genotype in both men and women.

### 2.2. Dietary Intake according to TAS2R4 Genotype

Nutrient intake and food consumption according to the *TAS2R4* rs2233998 genotype are presented in Table 2. The percentage of energy intake from carbohydrates, fat, protein, and the intake of sugars and dietary fiber were not associated with genetic variants in men and women. Daily total energy intake for women was only marginally different among each genotype group (CC: 1815 ± 554 kcal; CT: 1915 ± 636 kcal; TT: 1912 ± 649 kcal; *p*-value = 0.0636). In both men and women, the consumption of most food groups was not associated with their genotype. However, a marginally significant difference according to genotype was observed only in the consumption of grains and grain products among men (CC: 824.9 ± 231.6 kcal, CT: 790.9 ± 226.4, TT: 806.8 ± 208.1; *p*-value = 0.0708). 

### 2.3. Clinical Parameters Associated with T2DM according to TAS2R4 Genotype

The associations of clinical parameters related to T2DM with the *TAS2R4* rs2233998 genotype are presented in Table 3. In women, fasting glucose levels (CC: 82.8 ± 7.4 mg/dL, CT: 81.3 ± 7.9 mg/dL, TT: 81.3 ± 7.7; *p*-value = 0.0151) and prevalence of insulin resistance (defined by homeostasis model assessment of insulin resistance >2) (CC: 21.5%, CT: 22.1%, TT: 29.5%; *p*-value = 0.0292) were different among different genotype groups. However, in men, various T2DM-related parameters were not associated with the genetic variants.

### 2.4. Associations between TAS2R4 Genotype and T2DM Incidence

During the mean follow-up period of 11.97 ± 3.57 years (54,475 person-years), 1082 new cases (568 in men and 514 in women) of T2DM were reported. The *TAS2R4* rs2233998 genotype was significantly associated with T2DM incidence (Table 4). In the fully adjusted models, which included age, area of residence, education level, smoking status, alcohol consumption, total physical activity, and family history of diabetes, women carrying the TT genotype exhibited a significantly higher incidence of T2DM than those carrying the CC genotype (hazard ratio [HR]: 1.48; 95% confidence interval [CI]: 1.13–1.93). In men, no association was observed between the *TAS2R4* rs2233998 genotype and T2DM incidence.

### 2.5. Interactions between TAS2R4 Genotype and Dietary Intake in Relation to T2DM Incidence

We further examined whether carbohydrates, sugars, vegetables, and fruits modified the association between the *TAS2R4* rs2233998 genotype and T2DM incidence (Table 5). The relationship of the *TAS2R4* variants with dietary intake (high in carbohydrates or sugars; low in vegetables and fruits) increased the risk of T2DM development in women. Compared with women carrying the CC genotype in the lowest tertile of carbohydrate intake, those carrying the TT genotype in the corresponding highest tertile exhibited an increased incidence of T2DM (HR: 2.08, 95% CI: 1.33–3.27, *P*_interaction_ = 0.5217). However, TT carriers in the highest tertile of sugar intake exhibited an increased future risk of T2DM (HR: 2.31, 95% CI: 1.53–3.51, *P*_interaction_ = 0.0422) than CC carriers in the lowest tertile. Compared with the women carrying the CC genotype in the highest tertile of vegetable consumption, those carrying the TT genotype in the corresponding lowest tertile exhibited an increased incidence of T2DM (HR: 1.55, 95% CI: 1.02–2.37, *P*_interaction_ = 0.1660), while CC carriers in the lowest tertile of fruit consumption exhibited an increased incidence of T2DM (HR: 1.62, 95% CI: 1.06–2.48, *P*_interaction_ = 0.6041). However, no significant association was observed between *TAS2R4* rs2233998 variants and dietary intake impacting the risk status of T2DM in men.

## 3. Discussion

In this prospective cohort study with a long-term follow-up of 16 years, we investigated the relationship between the variants of bitter taste receptor genes and the risk of developing T2DM. The study revealed a significant association between the *TAS2R4* rs2233998 genotype and the incidence of T2DM in women, which varied according to dietary intake. In the fully adjusted model, the TT genotype of *TAS2R4* rs2233998 was significantly associated with fasting glucose levels, prevalence of insulin resistance, and increased risk of T2DM incidence in Korean women.

The relationship between the bitter taste receptors and health status has been investigated previously. As the bitter taste receptors are involved in the regulation of food intake mechanisms, their detailed investigation can unravel the pathogenesis of metabolic disorders, such as obesity and T2DM [18]. A Sorbs cohort study consisting of populations from eastern Germany revealed a significant link between haplotypes of *TAS2R38*, the gene encoding a common bitter taste receptor, and parameters of glucose homeostasis in men without diabetes [19]. Dotson et al. [13] also reported that the haplotype of *TAS2R* was significantly associated with insulin and glucose homeostasis. Moreover, functionally impaired *TAS2R* can affect the risk of metabolic diseases by altering the taste-receptor-mediated response to chemostimuli in the gustatory and digestive systems. The bitter taste receptor, a G-protein coupled receptor that plays an important role in perceiving bitter taste, exists in various extra-oral locations, including the gastrointestinal and respiratory systems, as well as in the taste buds of the tongue [20]. Because sweet and umami taste receptors mainly play a role in detecting energy and amino acids in foods, they stimulate mechanisms that accept, digest, and absorb foods [21]. However, because the bitter taste receptor detects toxic substances that may harm the body, they reduce food absorption and the speed of gastric emptying when bitter taste is detected [22]. 

Bitter substances induce bitter taste receptors, which secrete glucagon-like peptide-1 (GLP-1). GLP-1 increases insulin secretion, reduces glucagon, and suppresses appetite, resulting in a decrease in blood glucose levels by activating α-gustducin, which is a subunit of G-protein. Jang et al. [23] found that GLP-1 deficiency due to defects in α-gustducin expression might lead to a disturbance in glucose homeostasis, including the dysregulation of plasma insulin and glucose levels in α-gustducin-null mice. Another study reported that gut-expressed bitter taste receptors stimulate the secretion of GLP-1 in endocrine cells, which leads to the secretion of insulin in the pancreas and affects the reduction of blood glucose levels in mice with diabetes [24]. The expression of bitter taste receptors is associated with the secretion of various hormones, such as GLP-1, ghrelin, cholecystokinin, and peptide YY, which are involved in controlling appetite and blood glucose levels. This suggests that bitter taste and its receptors are potential therapeutic targets for T2DM [18,25,26].

Dietary factors are one of the main contributors to the prevalence of diabetes. Previous cohort studies have reported that the intake of carbohydrates, vegetables, and fruits is related to the incidence of T2DM. A recent meta-analysis that included 18 cohort studies revealed a relationship between carbohydrate intake and the risk of T2DM based on dose-response analysis; they reported that at 50% energy intake from carbohydrates, the risk of T2DM was the lowest (HR: 0.95, 95% CI: 0.90–0.99), whereas the risk was high when the intake of energy from carbohydrates was higher than 70% (HR: 1.18, 95% CI: 1.03–1.35) [27]. The intake of vegetables and fruits, unlike carbohydrates, decreases the risk of T2DM. A cohort study consisting of 6961 Swedish adults aged 35–56 years reported that men with a high total intake of fruit and vegetables exhibited a lower incidence of T2DM than those with low intake [28]. Moreover, a meta-analysis involving 22 studies reported that the total intake of fruits and green leafy, yellow, and cruciferous vegetables was associated with a decreased incidence of T2DM [29].

Many studies have investigated the independent effects of dietary and genetic factors to reveal the etiology of T2DM. However, since T2DM is a multifactorial disease [30], it is important to consider the effects of gene–diet interactions on the development of T2DM [31]. Moreover, environmental and genetic factors affect taste sensitivity [32]. Thus, in this study, we investigated the associations between *TAS2R4* gene variants and T2DM incidence and the effect of the dietary intake of carbohydrates, sugars, vegetables, and fruits on their association. The women carrying the TT genotype with high intakes of carbohydrates or sugars exhibited 2.08 times (HR: 2.08, 95% CI: 1.33–3.27 for carbohydrates) and 2.31 times (HR: 2.31, 95% CI: 1.53–3.51 for sugars), respectively, higher incidence of T2DM than the women carrying the CC genotype with lower intakes of carbohydrates or sugars. Moreover, the women carrying the TT genotype with low intakes of vegetables or fruits exhibited 1.55 times (HR: 1.55, 95% CI: 1.02–2.37 for vegetables) and 1.62 times (HR: 1.62, 95% CI: 1.06–2.48 for fruits), respectively, higher incidence of T2DM than CC carriers with high intakes of vegetables or fruits. Our results demonstrated that the association between the *TAS2R4* rs2233998 genotype and T2DM incidence was more pronounced in individuals with high intakes of carbohydrates or sugar and low intakes of vegetables and fruits.

This study revealed the differences in the effect of the interaction between genetic variations in bitter taste receptors and dietary intake on the incidence of T2DM by sex. Although the underlying biological mechanism is still unclear, the sex-differential effects of the interaction between the bitter taste receptor gene variants and dietary intake on T2DM based on sex can be partly explained by the following reasons. First, the sensitivity and preference for bitter taste vary with sex. Women have a higher sensitivity to bitter taste, detect bitter taste more strongly [33], and experience more changes in their sensitivity to bitter taste due to changes in their hormone levels compared to men. Moreover, high sensitivity to bitter taste is associated with a high preference for sweet taste [34], and a low sensitivity to sweet taste has been reported in individuals with obesity or diabetes [35]. Second, the sex-specific relationship between bitter taste receptor gene variants and the incidence of T2DM can be explained by the difference in the action of endogenous sex hormones between sexes. In both men and women, testosterone level is significantly associated with the risk of T2DM [36]; however, estradiol is involved in the incidence of diabetes only in women [37].

As the results of this study are based on the residents of two areas of Korea, Ansan (urban) and Ansung (rural), these results cannot be applied to the entire Korean population. Moreover, although various single nucleotide polymorphisms (SNPs) of *TAS2R* family genes have been reported in previous studies, this study analyzed only a limited number of SNPs of the *TAS2R4* gene that had a significant association with T2DM-related phenotypes. Despite this limitation, our study investigated the effect of taste receptor gene variant–diet interaction on T2DM incidence in Korean adults based on a relatively large cohort with a long follow-up period of 16 years. Moreover, the dietary information used in this study was collected using the food frequency questionnaire (FFQ), which was developed and validated to understand the usual dietary intake of the participants. Lastly, to the best of our knowledge, this is the first study to investigate the association between the genetic variation of bitter taste receptors and T2DM incidence and the effect of dietary intake on this association in the Korean population.

## 4. Materials and Methods

### 4.1. Data Source and Study Population

We used data from the Ansan-Ansung cohort study, which is part of the Korean Genome and Epidemiology Study (KoGES) conducted by the Korea National Institute of Health. To explore the environmental and genetic factors affecting the prevalence of chronic diseases in Koreans, the Ansan-Ansung cohort study began recruiting 10,030 Korean adults aged 40–69 years in 2001, and the participants were followed up biennially. Detailed information has been provided elsewhere [38]. Trained interviewers collected information regarding demographic factors, dietary intake, lifestyle, and the history of disease and medication of the participants using structured questionnaires. Blood and urine samples were collected from the patients during their visits to the examination center and were analyzed in a certified laboratory using a standard protocol.

Among the 10,030 participants aged 40–69 years at baseline, participants with (i) T2DM (*n* = 1098) or any type of cancer (*n* = 192) at baseline, (ii) no follow-up visits after baseline examination (*n* = 912), and (iii) no data on *TAS2R4* rs2233998 genotype (*n* = 2853) or dietary intake (*n* = 253) were excluded. Of the remaining 4722 participants, those who had implausible energy intake (<500 or >5000 kcal/day, *n* = 66) or missing information on covariates (*n* = 104) were also excluded. Finally, 4552 participants (2181 men and 2371 women) were included in the study. All methods and protocols were conducted in accordance with relevant institutional guidelines and regulations, and all participants provided written informed consent. This study was reviewed and approved by the Institutional Review Board of the Korea National University of Education (IRB No. KNUE-202011-BMBR-0102-01).

### 4.2. Genotyping and SNP Selection

Genotype data were obtained from the Korea Biobank Arrary (KoreanChip) containing 833,535 SNPs, which were generated by the Center for Genome Science, Korea National Institute of Health, to build a large-scale genomic data repository for the Korean population [39]. The sample exclusion criteria for standard quality control were as follows: Hardy–Weinberg equilibrium *p*-value < 1.0 × 10^−6^, missing call rate > 5%, or minor allele frequency < 0.01. Among the 74 SNPs reported in the *TAS2R* family, the *TAS2R4* rs2233998 genotype exhibits a significant association with T2DM-related phenotypes; therefore, it was investigated in this study.

### 4.3. Dietary Assessment

Information regarding the dietary intake of the participants was obtained at baseline by well-trained interviewers using a validated semi-quantitative FFQ. The FFQ, developed to evaluate usual food and nutrient intake of KoGES participants, consists of 103 food items and beverages covering approximately 85% of the selected nutrients [40,41]. The FFQ includes the following frequency categories: never/rarely, once a month, two–three times a month, one–two times a week, three–four times a week, five–six times a week, once a day, twice a day, or more than three times a day; portion size was divided into the following three categories: small, medium, or large. Daily food and nutrient intake was estimated by multiplying the consumption frequency of each food and beverage item by its energy and nutrient content in one portion size, and all these values were added. Nutritional information for all food and beverage items included in the FFQ was calculated by referring to the Korean Food Composition Table [42]. In this study, carbohydrate, sugar, vegetable, and fruit intakes were assessed based on sex-specific tertiles.

### 4.4. Definition of T2DM

Participants were identified as new cases of T2DM if they exhibited one of the following characteristics: fasting blood glucose level measured after at least 8 h ≥ 126 mg/dL and blood glucose level after 2 h 75 g oral glucose tolerance test ≥ 200 mg/dL; newly diagnosed with T2DM; or under treatment with insulin or oral antidiabetic drugs during or between the follow-up examinations based on the criteria of the World Health Organization [43] and the American Diabetes Association [44]. The primary endpoint was the first occurrence of T2DM, and the participants without new T2DM events were censored on the date of the last follow-up examination.

### 4.5. Statistical Analyses

All statistical analyses were conducted using PLINK (version 1.9, https://www.coggenomics.org/plink/1.9, accessed on 15 May 2022) and SAS (version 9.4, SAS Institute Inc., Cary, NC, USA). T2DM-associated SNPs in the *TAS2R* family genes and genotype frequencies were identified with logistic regression analysis using PLINK. We tested three different genetic models (additive, dominant, and recessive), and an additive genetic model (CC, CT, vs. TT) was selected for further analyses. Baseline characteristics of study participants according to *TAS2R4* rs2233998 genotype were compared using the Mantel–Haenszel chi-square test for categorical variables and one-way analysis of variance (ANOVA) for continuous variables. All data analyses were performed separately for men and women. Multivariable Cox proportional hazard models were used to calculate adjusted HRs and 95% CIs for T2DM incidence based on genotypes and dietary intake. The following potential covariates were included in the analytical models: age (years, continuous), area of residence (Ansan or Ansung), education level (≤elementary school, middle/high school, or ≥college), smoking status (never, past, or current smoker), alcohol consumption (g/d, continuous), BMI (kg/m^2^, continuous), total physical activity (metabolic equivalent task-hour/week, continuous), and family history of diabetes (yes or no). A two-sided *p*-value of <0.05 was considered significant.

## 5. Conclusions

The present study revealed significant associations between the *TAS2R4* rs2233998 genotype and the incidence of T2DM among women. We also found that the association between taste receptor gene variants and T2DM incidence varied with dietary intake. Women carrying the TT genotype with excessive intake of carbohydrate/sugars and inadequate intake of fruits/vegetables exhibited an increased incidence of T2DM compared to the women carrying the CC genotype. This study suggests that both dietary intake and the genotype of the bitter taste receptor gene influence the risk of developing T2DM in Korean women. Our findings elucidate the importance of an integrated approach involving taste receptor gene variants and dietary intake in establishing strategies for preventing T2DM.

## Figures and Tables

**Table 1:** General characteristics of the participants at baseline according to *TAS2R4* rs2233998 genotype.

	Men				Women			
	rs2233998 Genotype			*p*-Value	rs2233998 Genotype			*p*-Value
	CC	CT	TT		CC	CT	TT	
	(*n* = 1029, 47.18%)	(*n* = 937, 42.96%)	(*n* = 215, 9.86%)		(*n* = 1037, 43.74%)	(*n* = 1080, 45.55%)	(*n* = 254, 10.71%)	
Age, yr	50.7 ± 8.2	50.5 ± 8.3	50.2 ± 8.2	0.6193	51.9 ± 8.6	51.0 ± 8.4	52.3 ± 8.7	0.0169
Area of residence				0.5071				0.6872
Ansung (rural)	404 (39.3)	374 (39.9)	90 (41.9)		549 (52.9)	561 (51.9)	142 (55.9)	
Ansan (urban)	625 (60.7)	563 (60.1)	125 (58.1)		488 (47.1)	519 (48.1)	112 (44.1)	
Education level				0.9328				0.6399
≤elementary school	167 (16.2)	155 (16.5)	32 (14.9)		448 (43.2)	419 (38.8)	112 (44.1)	
middle/high school	622 (60.5)	570 (60.8)	132 (61.4)		526 (50.7)	589 (54.5)	131 (51.6)	
≥college	240 (22.6)	212 (22.6)	51 (23.7)		63 (6.1)	72 (6.7)	11 (4.3)	
Smoking status				0.3963				0.2090
Never	218 (21.2)	193 (20.6)	37 (17.2)		1008 (97.2)	1031 (95.5)	245 (96.5)	
Past	337 (32.8)	295 (31.5)	79 (36.7)		6 (0.6)	16 (1.5)	2 (0.8)	
Current	474 (46.1)	449 (47.9)	99 (46.1)		23 (2.2)	33 (3.1)	7 (2.8)	
Alcohol consumption, g/d	17.3 ± 25.2	18.8 ± 28.0	18.2 ± 29.8	0.4719	1.0 ± 4.3	1.4 ± 5.5	1.4 ± 4.6	0.1498
Body mass index, kg/m^2^	24.3 ± 2.8	24.3 ± 3.0	24.2 ± 2.8	0.7566	24.7 ± 3.1	24.7 ± 3.1	24.5 ± 3.2	0.6736
Total physical activity, MET-h/wk	164.2 ± 99.3	168.8 ± 104.4	169.1 ± 114.9	0.5724	168.9 ± 106.8	162.1 ± 98.5	168.2 ± 99.1	0.2882
Family history of diabetes				0.6285				0.4836
Yes	97 (9.4)	90 (9.6)	23 (10.7)		123 (11.9)	123 (11.4)	26 (10.2)	
No	932 (90.6)	847 (90.4)	192 (89.3)		914 (88.1)	957 (88.6)	228 (89.8)	

Values are numbers (percentage) for categorical variables and mean ± standard deviation for continuous variables. MET-h/wk, metabolic equivalent task-hour/week.

**Table 2:** Nutrient and food-group intake according to *TAS2R4* rs2233998 genotype.

	Men				Women			
	rs2233998 Genotype			*p*-Value	rs2233998 Genotype			*p*-Value
	CC	CT	TT		CC	CT	TT	
Energy, kcal/d	2049 ± 536	2027 ± 578	2062 ± 563	0.3902	1815 ± 544	1915 ± 636	1912 ± 649	0.0636
Nutrient intake								
% Energy from carbohydrates	72.9 ± 6.7	72.4 ± 6.7	72.4 ± 6.6	0.5604	75.5 ± 6.6	75.0 ± 6.9	74.8 ± 7.2	0.3545
% Energy from fat	14.2 ± 5.0	14.5 ± 5.0	14.5 ± 4.9	0.6854	11.9 ± 4.8	12.3 ± 5.1	12.4 ± 5.3	0.3275
% Energy from total protein	12.9 ± 2.1	13.1 ± 2.3	13.1 ± 2.3	0.4464	12.6 ± 2.3	12.7 ± 2.4	12.8 ± 2.4	0.5907
Sugar, g/d	33.2 ± 15.4	35.3 ± 16.4	34.7 ± 16.1	0.2019	40.3 ± 21.8	40.6 ± 22.2	40.9 ± 21.8	0.9178
Dietary fiber, g/d	12.3 ± 4.7	12.9 ± 5.2	12.9 ± 5.1	0.2903	15.3 ± 6.1	14.9 ± 6.5	15.2 ± 6.2	0.5290
Food-group consumption, g/d								
Grains and grain products	824.9 ± 231.6	790.9 ± 226.4	806.8 ± 208.1	0.0708	722.1 ± 216.3	748.9 ± 258.0	743.6 ± 254.6	0.3123
Vegetables	115.4 ± 96.0	117.6 ± 103.6	118.2 ± 97.5	0.9322	130.7 ± 128.1	129.4 ± 113.6	132.0 ± 137.5	0.8934
Fruits	192.3 ± 225.7	219.2 ± 252.9	215.0 ± 249.8	0.3582	283.4 ± 296.8	306.5 ± 350.9	305.6 ± 376.4	0.6326
Meat, eggs, and fish	121.6 ± 86.9	126.0 ± 82.2	130.0 ± 89.7	0.3445	85.8 ± 63.6	97.9 ± 79.3	97.9 ± 95.2	0.1005
Milk and dairy products	100.6 ± 119.2	104.2 ± 138.6	104.1 ± 134.3	0.9341	106.8 ± 124.1	116.5 ± 134.1	121.1 ± 150.3	0.3302
Soft drinks	29.5 ± 49.7	32.9 ± 56.9	31.6 ± 57.3	0.7030	14.5 ± 48.5	19.1 ± 56.2	17.6 ± 53.2	0.4602
Sweets and oils	11.2 ± 10.9	11.5 ± 11.4	11.4 ± 10.9	0.9317	7.2 ± 8.2	7.3 ± 8.8	7.2 ± 8.5	0.9932

*p*-values were obtained from the multiple linear regression.

**Table 3:** Clinical characteristics of the participants at baseline according to *TAS2R4* rs2233998 genotype.

	Men				Women			
	rs2233998 Genotype			*p*-Value	rs2233998 Genotype			*p*-Value
	CC	CT	TT		CC	CT	TT	
Fasting glucose level (mg/dL) (*n* = 2175, 2362)	84.0 ± 9.7	85.5 ± 9.3	85.1 ± 9.0	0.1141	82.8 ± 7.4	81.3 ± 7.9	81.3 ± 7.7	0.0151
2 h glucose level (mg/dL) (*n* = 2169, 2356)	107.6 ± 30.1	111.8 ± 32.0	113.0 ± 31.3	0.0721	121.2 ± 29.5	117.6 ± 28.2	118.4 ± 29.1	0.2045
Fasting insulin level (uIU/mL) (*n* = 2175, 2362)	6.5 ± 3.3	6.9 ± 4.0	7.1 ± 4.2	0.1725	8.2 ± 5.6	7.9 ± 5.0	7.8 ± 4.2	0.3746
HOMA-IR (*n* = 2175, 2362)	1.4 ± 0.8	1.5 ± 0.9	1.5 ± 0.9	0.1579	1.7 ± 1.2	1.6 ± 1.0	1.6 ± 0.9	0.1987
HbA1c concentration (%)	5.5 ± 0.3	5.6 ± 0.4	5.6 ± 0.4	0.1126	5.6 ± 0.4	5.6 ± 0.4	5.6 ± 0.4	0.5993
Impaired fasting glucose level [*n* (%)]	62 (6.0)	70 (7.5)	18 (8.4)	0.1226	19 (1.8)	30 (2.8)	5 (2.0)	0.4240
Insulin resistance [*n* (%)]	216 (21.0)	185 (19.7)	41 (19.1)	0.4157	223 (21.5)	239 (22.1)	75 (29.5)	0.0292
HbA1c 5.7–6.4% [*n* (%)]	368 (35.8)	346 (36.9)	75 (34.9)	0.9204	351 (33.9)	352 (32.6)	90 (35.4)	0.9537

*p*-values were obtained from the multiple linear regression. HOMA-IR, homeostasis model assessment of insulin resistance; HbA1C, glycated hemoglobin.

**Table 4:** Adjusted hazard ratios (with 95% confidence intervals) for type 2 diabetes mellitus according to *TAS2R4* rs2233998 genotype.

	Men				Women			
	rs2233998 Genotype			*p*-Value	rs2233998 Genotype			*p*-Value
	CC	CT	TT		CC	CT	TT	
Person-years	11,962	10,891	2535		12,921	13,141	3025	
Incident cases (*n*)	263	248	57		215	229	70	
Rate per 1000 person-years	22.0	22.8	22.5		16.6	17.4	23.1	
	HR (95% CI)	HR (95% CI)	HR (95% CI)		HR (95% CI)	HR (95% CI)	HR (95% CI)	
T2DM incidence	Ref.	1.01 (0.85–1.21)	1.05 (0.78–1.39)	0.9536	Ref.	1.07 (0.89–1.30)	1.48 (1.13–1.93)	0.0182

Models were adjusted for age (years, continuous), area of residence (Ansan or Ansung), education level (≤elementary school, middle/high school, or ≥college), smoking status (never, past, or current smoker), alcohol consumption (g/d, continuous), body mass index (kg/m^2^, continuous), total physical activity (metabolic equivalent task-hour/week, continuous), and family history of diabetes (yes or no). HR, hazard ratio; 95% CI, 95% confidence interval; T2DM, type 2 diabetes mellitus.

**Table 5:** Adjusted hazard ratios (with 95% confidence intervals) for type 2 diabetes mellitus according to *TAS2R4* rs2233998 genotype and dietary intake.

	Men				Women			
	rs2233998 Genotype			*p*-Value	rs2233998 Genotype			*p*-Value
	CC	CT	TT		CC	CT	TT	
	HR (95% CI)	HR (95% CI)	HR (95% CI)		HR (95% CI)	HR (95% CI)	HR (95% CI)	
Carbohydrates				0.6875				0.5217
T1	ref.	0.99 (0.74–1.33)	1.25 (0.77–2.01)		ref.	1.24 (0.89–1.74)	1.35 (0.79–2.31)	
T2	0.95 (0.70–1.27)	0.91 (0.67–1.23)	0.61 (0.34–1.09)		1.17 (0.83–1.64)	1.12 (0.80–1.58)	1.36 (0.84–2.20)	
T3	0.90 (0.66–1.22)	0.99 (0.72–1.35)	1.19 (0.75–1.90)		1.07 (0.75–1.52)	1.12 (0.79–1.58)	2.08 (1.33–3.27)	
Sugars				0.6258				0.0422
T1	ref.	1.02 (0.76–1.38)	0.85 (0.49–1.50)		ref.	1.15 (0.83–1.59)	1.45 (0.84–2.51)	
T2	1.04 (0.78–1.39)	1.02 (0.75–1.38)	0.82 (0.49–1.38)		1.11 (0.80–1.55)	1.32 (0.96–1.83)	1.15 (0.70–1.88)	
T3	0.83 (0.61–1.12)	0.87 (0.64–1.18)	1.29 (0.83–2.00)		1.16 (0.84–1.62)	1.04 (0.75–1.46)	2.31 (1.53–3.51)	
Vegetables				0.3644				0.1660
T1	1.09 (0.81–1.47)	0.87 (0.63–1.19)	1.16 (0.70–1.91)		0.81 (0.57–1.13)	0.97 (0.70–1.35)	1.55 (1.02–2.37)	
T2	1.11 (0.82–1.50)	1.22 (0.91–1.63)	1.09 (0.67–1.77)		1.00 (0.72–1.38)	0.97 (0.70–1.35)	1.36 (0.83–2.24)	
T3	ref.	1.15 (0.85–1.55)	1.10 (0.66–1.82)		ref.	1.06 (0.77–1.46)	1.17 (0.70–1.94)	
Fruits				0.7229				0.6041
T1	1.11 (0.83–1.50)	1.05 (0.78–1.43)	1.45 (0.92–2.27)		0.92 (0.66–1.28)	0.99 (0.72–1.37)	1.62 (1.06–2.48)	
T2	1.10 (0.81–1.48)	1.05 (0.77–1.43)	1.02 (0.62–1.66)		0.97 (0.70–1.34)	1.04 (0.76–1.44)	1.12 (0.66–1.88)	
T3	ref.	1.15 (0.85–1.56)	0.85 (0.47–1.56)		ref.	1.07 (0.77–1.48)	1.48 (0.91–2.41)	

Data are presented as adjusted hazard ratios (HRs) and 95% confidence intervals (CIs). Models were adjusted for age (years, continuous), area of residence (Ansan or Ansung), education level (≤elementary school, middle/high school, or ≥college), smoking status (never, past, or current smoker), alcohol consumption (g/d, continuous), body mass index (kg/m^2^, continuous), total physical activity (metabolic equivalent task-hour/week, continuous), and family history of diabetes (yes or no). HR, hazard ratio; 95% CI, 95% confidence interval; T, tertile.

## Data Availability

The dataset used in this study (Ansan-Ansung cohort study of the KoGES) was obtained after reviewing and evaluating the research plan of the Korea National Institute of Health, Korea Disease Control and Prevention Agency (http://nih.go.kr/contents.es?mid=a50401010400, accessed on 7 March 2022).

## References

[1] International Diabetes Federation (2021). IDF Diabetes Atlas.

[2] Institute for Health Metrics and Evaluation. https://www.healthdata.org/south-korea.

[3] Jung C.H., Son J.W., Kang S., Kim W.J., Kim H.-S., Kim H.S., Seo M., Shin H.J., Lee S.S., Jeong S.J. (2021). Diabetes fact sheets in Korea, 2020: An appraisal of current status. Diabetes Metab. J..

[4] Sladek R., Rocheleau G., Rung J., Dina C., Shen L., Serre D., Boutin P., Vincent D., Belisle A., Hadjadj S. (2007). A genome-wide association study identifies novel risk loci for type 2 diabetes. Nature.

[5] Cho Y.S., Chen C.H., Hu C., Long J., Hee Ong R.T., Sim X., Takeuchi F., Wu Y., Go M.J., Yamauchi T. (2012). Meta-analysis of genome-wide association studies identifies eight new loci for type 2 diabetes in east Asians. Nat. Genet..

[6] Go M.J., Hwang J.Y., Park T.J., Kim Y.J., Oh J.H., Kim Y.J., Han B.G., Kim B.J. (2014). Genome-wide association study identifies two novel Loci with sex-specific effects for type 2 diabetes mellitus and glycemic traits in a Korean population. Diabetes Metab. J..

[7] Kim Y.J., Go M.J., Hu C., Hong C.B., Kim Y.K., Lee J.Y., Hwang J.Y., Oh J.H., Kim D.J., Kim N.H. (2011). Large-scale genome-wide association studies in East Asians identify new genetic loci influencing metabolic traits. Nat. Genet..

[8] Drewnowski A. (1997). Taste preferences and food intake. Annu. Rev. Nutr..

[9] Garcia-Bailo B., Toguri C., Eny K.M., El-Sohemy A. (2009). Genetic variation in taste and its influence on food selection. OMICS J. Integr. Biol..

[10] Fernández-Carrión R., Sorlí J.V., Coltell O., Pascual E.C., Ortega-Azorín C., Barragán R., Giménez-Alba I.M., Alvarez-Sala A., Fitó M., Ordovas J.M. (2021). Sweet taste preference: Relationships with other tastes, liking for sugary foods and exploratory genome-wide association analysis in subjects with metabolic syndrome. Biomedicines.

[11] Ramos-Lopez O., Panduro A., Martinez-Lopez E., Roman S. (2016). Sweet taste receptor *TAS1R2* polymorphism (Val191Val) is associated with a higher carbohydrate intake and hypertriglyceridemia among the population of West Mexico. Nutrients.

[12] Di Bona D., Malovini A., Accardi G., Aiello A., Candore G., Ferrario A., Ligotti M.E., Maciag A., Puca A.A., Caruso C. (2021). Taste receptor polymorphisms and longevity: A systematic review and meta-analysis. Aging Clin. Exp. Res..

[13] Dotson C.D., Zhang L., Xu H., Shin Y.K., Vigues S., Ott S.H., Elson A.E., Choi H.J., Shaw H., Egan J.M. (2008). Bitter taste receptors influence glucose homeostasis. PLoS ONE.

[14] Choi J.H., Lee J., Choi I.J., Kim Y.W., Ryu K.W., Kim J. (2016). Genetic variation in the *TAS2R38* bitter taste receptor and gastric cancer risk in Koreans. Sci. Rep..

[15] Choi J.H., Lee J., Oh J.H., Chang H.J., Sohn D.K., Shin A., Kim J. (2017). Variations in the bitterness perception-related genes *TAS2R38* and *CA6* modify the risk for colorectal cancer in Koreans. Oncotarget.

[16] Baharuddin A., Sharifudin M.S. (2015). The impact of geographical location on taste sensitivity and preference. Int. Food Res. J..

[17] Ahrens W. (2015). Sensory taste preferences and taste sensitivity and the association of unhealthy food patterns with overweight and obesity in primary school children in Europe—A synthesis of data from the IDEFICS study. Flavour.

[18] Cummings D.E., Overduin J. (2007). Gastrointestinal regulation of food intake. J. Clin. Investig..

[19] Keller M., Liu X., Wohland T., Rohde K., Gast M.T., Stumvoll M., Kovacs P., Tönjes A., Böttcher Y. (2013). *TAS2R38* and its influence on smoking behavior and glucose homeostasis in the German Sorbs. PLoS ONE.

[20] Lu P., Zhang C.H., Lifshitz L.M., ZhuGe R. (2017). Extraoral bitter taste receptors in health and disease. J. Gen. Physiol..

[21] Yarmolinsky D.A., Zuker C.S., Ryba N.J.P. (2009). Common sense about taste: From mammals to insects. Cell.

[22] Dong D., Jones G., Zhang S. (2009). Dynamic evolution of bitter taste receptor genes in vertebrates. BMC Ecol. Evol..

[23] Jang H.J., Kokrashvili Z., Theodorakis M.J., Carlson O.D., Kim B.J., Zhou J., Kim H.H., Xu X., Chan S.L., Juhaszova M. (2007). Gut-expressed gustducin and taste receptors regulate secretion of glucagon-like peptide-1. Proc. Natl. Acad. Sci. USA.

[24] Kim K.S., Egan J.M., Jang H.J. (2014). Denatonium induces secretion of glucagon-like peptide-1 through activation of bitter taste receptor pathways. Diabetologia.

[25] Xie C., Wang X., Young R.L., Horowitz M., Rayner C.K., Wu T. (2018). Role of intestinal bitter sensing in enteroendocrine hormone secretion and metabolic control. Front. Endocrinol..

[26] Batterham R.L., Cowley M.A., Small C.J., Herzog H., Cohen M.A., Dakin C.L., Wren A.M., Brynes A.E., Low M.J., Ghatei M.A. (2002). Gut hormone PYY3-36 physiologically inhibits food intake. Nature.

[27] Hosseini F., Jayedi A., Khan T.A., Shab-Bidar S. (2022). Dietary carbohydrate and the risk of type 2 diabetes: An updated systematic review and dose–response meta-analysis of prospective cohort studies. Sci. Rep..

[28] Barouti A.A., Tynelius P., Lager A., Björklund A. (2022). Fruit and vegetable intake and risk of prediabetes and type 2 diabetes: Results from a 20-year long prospective cohort study in Swedish men and women. Eur. J. Nutr..

[29] Wang P.Y., Fang J.C., Gao Z.H., Zhang C., Xie S.Y. (2016). Higher intake of fruits, vegetables or their fiber reduces the risk of type 2 diabetes: A meta-analysis. J. Diabetes Investig..

[30] Lee K.W., Woo H.D., Cho M.J., Park J.K., Kim S.S. (2019). Identification of dietary patterns associated with incidence of hyperglycemia in middle-aged and older Korean adults. Nutrients.

[31] Qin J., Li Y., Cai Z., Li S., Zhu J., Zhang F., Liang S., Zhang W., Guan Y., Shen D. (2012). A metagenome-wide association study of gut microbiota in type 2 diabetes. Nature.

[32] Navarro-Allende A., Khataan N., El-Sohemy A. (2008). Impact of genetic and environmental determinants of taste with food preferences in older adults. J. Nutr. Elder..

[33] Hyde R.J., Feller R.P. (1981). Age and sex effects on taste of sucrose, NaCl, citric acid and caffeine. Neurobiol. Aging.

[34] Bawajeeh A.O., Albar S.A., Zhang H., Zulyniak M.A., Evans C.E., Cade J.E. (2020). Impact of taste on food choices in adolescence—Systematic review and meta-analysis. Nutrients.

[35] Kumari B.G., Kumar Z.N. (2020). Evaluation of sweet taste sensitivity in type-II Diabetes Mellitus patients. Int. J. Clin. Biomed. Res..

[36] Mather K., Kim C., Christophi C., Aroda V., Knowler W., Edelstein S., Florez J., Labrie F., Kahn S., Goldberg R.B. (2015). Steroid sex hormones, sex hormone–binding globulin, and diabetes incidence in the diabetes prevention program. J. Clin. Endocrinol. Metab..

[37] Goodman-Gruen D., Barrett-Connor E. (2000). Sex differences in the association of endogenous sex hormone levels and glucose tolerance status in older men and women. Diabetes Care.

[38] Kim Y., Han B., KoGES Group (2016). Cohort profile: The Korean genome and epidemiology study (KoGES) Consortium. Int. J. Epidemiol..

[39] Moon S., Kim Y.J., Han S., Hwang M.Y., Shin D.M., Park M.Y., Lu Y., Yoon K., Jang H.M., Kim Y.K. (2019). The Korea Biobank Array: Design and identification of coding variants associated with blood biochemical traits. Sci. Rep..

[40] Ahn Y., Kwon E., Shim J., Park M., Joo Y., Kimm K., Park C., Kim D.H. (2007). Validation and reproducibility of food frequency questionnaire for Korean genome epidemiologic study. Eur. J. Clin. Nutr..

[41] Ahn Y., Lee J.E., Paik H.Y., Lee H.K., Jo I., Kim K. (2003). Development of a semi-quantitative food frequency questionnaire based on dietary data from the Korea National Health and Nutrition Examination Survey. Nutr. Sci..

[42] National Institute of Agricultural Sciences (KR) (2017). Korean Standard Food Composition Table, 9th Revision.

[43] World Health Organization (2006). Definition and Diagnosis of Diabetes Mellitus and Intermediate Hyperglycaemia: Report of a WHO/IDF Consultation. https://apps.who.int/iris/bitstream/handle/10665/43588/9241594934_eng.pdf.

[44] American Diabetes Association (2014). Diagnosis and classification of diabetes mellitus. Diabetes Care.

